# Research progress of surface-enhanced Raman scattering technology in tumor liquid biopsy

**DOI:** 10.3389/fmolb.2026.1747626

**Published:** 2026-04-23

**Authors:** Yuanchao Cao, Dingyu Rao, Defa Huang, Xiaoqiang Xu, Yilei Zhang, Chunfa Xie, Shulin Li, Zhixian Tang, Shenyu Zhu, Chuan Yao

**Affiliations:** 1 Department of Cardiothoracic Surgery, The Affiliated Hospital of Jiujiang University, Jiujiang, China; 2 Department of Thoracic Surgery, First Affiliated Hospital of Gannan Medical University, Ganzhou, China; 3 Laboratory Medicine, The First Affiliated Hospital of Gannan Medical University, Ganzhou, China; 4 The First Clinical College, Gannan Medical University, Ganzhou, China

**Keywords:** exosome, liquid biopsy, surface-enhanced Raman scattering, tumor diagnosis, tumor markers

## Abstract

Surface-Enhanced Raman Scattering technology has shown broad application potential in tumor diagnosis due to its excellent sensitivity and specificity. With the increasing demand for early tumor detection and precision medicine, SERS has been widely applied in the detection of tumor markers, particularly in liquid biopsy. This review systematically summarizes the recent advances of SERS in detecting key liquid biopsy biomarkers (exosomes, CTCs, ctDNA), critically analyzes its advantages in sensitivity and multiplexing, and discusses the main challenges for clinical translation, including signal interference in complex biological environments and the need for improved quantitative accuracy. Future perspectives integrating AI and multimodal strategies are also outlined to guide the development of precision oncology tools.

## 1 Introduction

As a powerful spectroscopic analysis technique, Surface-Enhanced Raman Scattering (SERS) technology enables rapid, non-destructive, and high-sensitivity detection of biological samples by enhancing molecular Raman scattering signals, and has gradually become an important detection method in the biomedical field. The core advantages of SERS lie in its high specificity and sensitivity to molecular structures. It enables single-molecule-level detection in complex biological environments, possesses strong photobleaching resistance, and is suitable for multimodal imaging and dynamic monitoring (Va et al., 2025; Wu et al., 2024). In recent years, with the rapid development of nanomaterials and nanotechnology, the design of SERS substrates based on magnetic-plasmonic composite nanomaterials has been continuously optimized. These materials not only enhance the electromagnetic field effect but also integrate the capabilities of enriching, separating and selectively identifying biomolecules, which greatly improves the applicability and flexibility of SERS in biological detection (Wei and Liu, 2025). In addition, gold nanomaterials have become an important component in SERS probes due to their excellent surface plasmon resonance effect, tunable structural morphology, and good biocompatibility, and are widely used in in vivo disease diagnosis and imaging (Wen et al., 2022). Notable progress has also been made in the application of SERS technology in molecular imaging. By virtue of highly sensitive and specific “nanotags”, it has enabled a variety of preclinical applications such as 2D/3D cell culture, tissue imaging, *in vivo* diagnosis, and surgical navigation, providing strong support for the early diagnosis and treatment of diseases (Lin et al., 2021). Notably, by combining machine learning and deep learning algorithms, the performance of SERS nanotags in liquid biopsies has been significantly enhanced, accelerating the process of their clinical translation (Va et al., 2025).

Early detection of tumors is of crucial significance for improving patients' survival rates and treatment outcomes. However, traditional detection methods such as imaging examinations, tissue biopsies, and blood tumor marker detection have limitations including insufficient sensitivity, high invasiveness, and high false-positive rates, making it difficult to meet the clinical demand for accurate diagnosis of early-stage tumors (Pepe et al., 2025; Shaik et al., 2023). For example, as important components of liquid biopsies, Circulating Tumor DNA (ctDNA) and Circulating Tumor Cells (CTCs) have provided new ideas for non-invasive detection. However, limitations such as their low abundance and insufficient detection sensitivity have not yet been fully resolved, which is particularly prominent in patients with early-stage tumors (Suh et al., 2021; Feng et al., 2022). Meanwhile, the insufficient specificity and sensitivity of existing tumor markers limit their application value in early screening (Savas and Coskun, 2025). Therefore, the development of detection technologies with high sensitivity, high specificity, and simple operation has become a research hotspot in the field of early tumor diagnosis.

SERS technology exhibits unique potential in the detection of tumor markers. By leveraging nanostructure-enhanced Raman signals, it enables ultra-sensitive detection of a variety of biomarkers, including proteins, nucleic acids (encompassing DNA and RNA), and small molecules, thereby overcoming limitations of traditional methods such as weak signals and significant background interference (Wu et al., 2024; Nanda et al., 2025). For instance, SERS-based immunoassay technology can utilize highly specific probes to identify tumor-associated mRNA and proteins, enabling simultaneous detection of multiple biomarkers and boasting broad prospects for clinical application (Yang et al., 2022). In addition, by combining with magnetic nanoparticles, core-shell structures, and multimodal imaging technology, SERS probes can achieve accurate capture and localization of tumor cells, providing an efficient tool for early tumor diagnosis (Wilson et al., 2020; Akgönüllü and Denizli, 2023). In the field of liquid biopsies, SERS technology enables sensitive detection of exosomes, circulating tumor DNA (ctDNA), and CTCs, thereby facilitating the early screening and dynamic monitoring of tumors (Li et al., 2021; Liu et al., 2022). Notably, the excellent signal enhancement capability and multiplex labeling capability of SERS technology endow it with significant advantages in the combined detection of multiple tumor markers and the analysis of complex biological samples, further advancing its translational application in clinical diagnosis (Eremina et al., 2023; Rodriguez-Nieves et al., 2024).

Liquid biopsy has emerged as a transformative approach in oncology, enabling non-invasive detection and monitoring of tumors through the analysis of biomarkers in body fluids such as blood, urine, and saliva. Key analytes including circulating tumor cells (CTCs), circulating tumor DNA (ctDNA), and exosomes provide a comprehensive molecular profile of the tumor, reflecting its heterogeneity and dynamic evolution. Compared to traditional tissue biopsy, liquid biopsy offers advantages of minimal invasiveness, repeatable sampling, and the potential for real-time monitoring of treatment response and disease progression. However, the clinical utility of liquid biopsy is hindered by the extreme low abundance of these biomarkers in circulation and the high background interference from complex biological matrices, necessitating detection technologies with ultra-high sensitivity and specificity.

In summary, leveraging its advantages of high sensitivity, high specificity, and non-invasiveness, SERS technology has become a research hotspot in the field of early tumor diagnosis. By integrating the design optimization of nanomaterials, the integration of intelligent algorithms, and the combination of multimodal imaging, SERS technology is expected to break through the bottlenecks of traditional detection methods, achieve efficient and accurate detection of tumor markers, and promote the development of clinical early tumor screening and personalized treatment (Kalligosfyri et al., 2024). In the future, with the deepening of clinical translation research and the continuous improvement of technology, SERS will play an even more important role in tumor diagnosis.

## 2 Basic principles and enhancement mechanisms of SERS technology

### 2.1 The working principle of SERS

#### 2.1.1 The working principle of SERS

**FIGURE 1:**
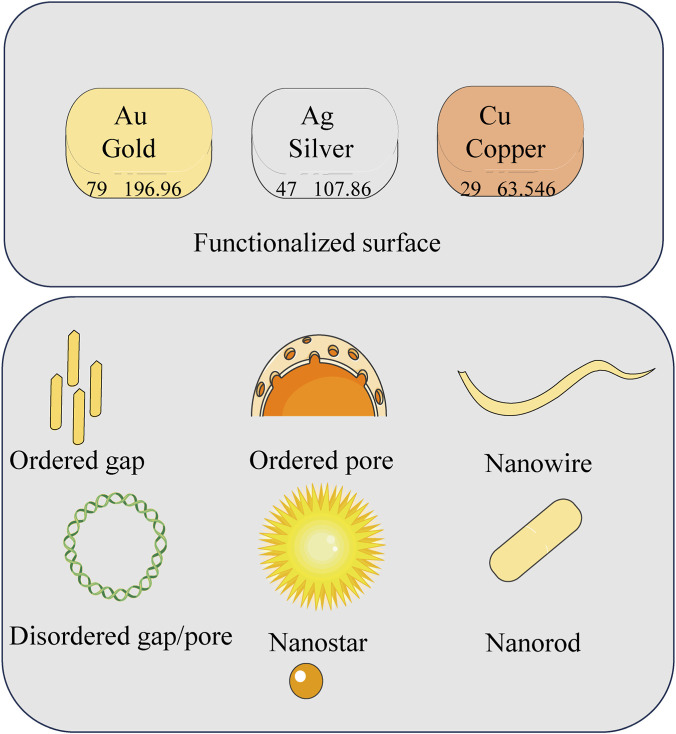
Common types of nanostructures.

Surface-Enhanced Raman Scattering (SERS) amplifies the inherently weak Raman signals of molecules adsorbed on or near nanostructured metal surfaces (Martínez-García and Martín-Cano, 2024). This massive enhancement (typically 10^4^–10^10^ fold) arises from the excitation of localized surface plasmon resonances (LSPR) in noble metal nanostructures (e.g., Au, Ag), which generate intense localized electromagnetic fields, known as “hot spots,” at sharp features or inter-particle gaps (Hossain et al., 2021; Huang et al., 2024). The intensity of the Raman signal is proportional to the fourth power of the local field enhancement (|E| (Wen et al., 2022)), making the engineering of these hot spots—through precise control of nanoparticle morphology, composition, and assembly—paramount for achieving high sensitivity (Guselnikova et al., 2022; Le Ru and Auguié, 2024). As shown in Figure 1, common SERS substrates include nanoparticles, nanorods, nanostars, nanowires, and various nanogap/nanopore structures, whose size, shape, and surface chemistry can be tailored to meet the requirements of specific biological applications (Chen and Qiu, 2025; Peng et al., 2024).

#### 2.1.2 Enhancement mechanisms

The extraordinary signal amplification in SERS arises from the synergistic effect of two primary mechanisms: electromagnetic enhancement (EM) and chemical enhancement (CM).

Electromagnetic Enhancement (EM): This is the dominant contribution, originating from the excitation of localized surface plasmon resonance (LSPR) in metallic nanostructures (e.g., Au, Ag) (Zhang et al., 2020).Upon resonant light excitation, collective electron oscillations generate intensely localized electromagnetic fields, known as “hotspots,” at nanoscale features such as sharp tips, edges, or inter-particle gaps. The Raman signal intensity is proportional to the fourth power of the local field enhancement (|E| (Wen et al., 2022)), making the engineering of these hotspots—through precise control of nanoparticle morphology, composition, and assembly—paramount for achieving high sensitivity. Recent research focuses on creating reproducible and dense hotspot arrays using anisotropic nanostructures (e.g., nanorods, nanostars) and complex 3D superstructures, including super-radiant modes in ordered arrays and sub-nanometer gap engineering (Seçkin et al., 2023; Mandelbaum et al., 2020; Bauman et al., 2022).

Chemical Enhancement (CM): This mechanism provides additional, molecule-specific signal enhancement through charge-transfer processes between the analyte molecules and the metal substrate (Song B. et al., 2025). When molecules chemically adsorb onto the surface, new electronic states may form, altering the molecular polarizability and effectively increasing its Raman scattering cross-section. While the enhancement factor from CM is typically lower (10–10^3^) compared to EM, it is crucial for substrate selectivity and can be tailored through surface functionalization with specific ligands or molecular imprinting.

Evolution of SERS Substrates: The pursuit of optimal performance has driven the evolution of SERS substrates from simple colloidal nanoparticles to sophisticated engineered materials (Wang et al., 2022; Zhang M. et al., 2025). Key developments include anisotropic particles for tunable LSPR, core-shell structures (e.g., Au@Ag) combining different material advantages, magnetic-plasmonic composites for integrated target enrichment and detection, and semiconductor-based substrates (e.g., MoS_2_, CoSe_2_) offering new charge-transfer pathways and improved stability through phase and interface engineering (Tiryaki et al., 2024). The choice of substrate is a critical decision that balances enhancement factor, reproducibility, biocompatibility, and functionalization capability for specific biomedical applications.

### 2.2 Application of SERS in liquid biopsy

Liquid biopsy is a non-invasive detection technology based on circulating tumor cells (CTCs), circulating tumor DNA (ctDNA), circulating RNA, exosomes, and other tumor-related biomarkers in body fluid samples (such as blood, urine, cerebrospinal fluid, etc*.*). Compared with traditional tissue biopsy, liquid biopsy has the advantages of simple sampling, minimal invasiveness, repeatable sampling, and the ability to reflect the systemic heterogeneity and dynamic changes of tumors. Therefore, it shows great potential in early tumor detection, monitoring of treatment response, and prognostic evaluation (Table 1).

**TABLE 1:** Comparison of SERS-based strategies for liquid biopsy biomarkers.

Target analyte	Sample type	SERS substrate design	Sensitivity/Specificity	Key advantage	Ref.
Exosomes (gastric cancer)	Serum	Gold nanostar@MoS2 nanocomposite	—	High stability, clinical applicability	Liu et al. (2024)
Exosomes (breast cancer)	Serum	Magnetic SERS platform (Fe3O4@Au)	91.67%/100%	Sequential isolation and detection	Li et al. (2021)
Exosomes (lung cancer)	Cell culture medium	Silver nanocube composite	95.23% accuracy	Deep learning (MobileNet V2) integration	Haldavnekar et al. (2022a)
ctDNA (DIPG)	—	Gold nanoparticles + enzymatic amplification	—	Single-base mutation identification	Sánchez-Castillo et al. (2024)
CTCs (breast cancer)	Blood	Multicolor SERS nanotags	—	Multiplex phenotyping	Dey, (2023)
Proteins (PSA-mediated PHI)	Serum	Dual-enhanced SERS immuno-nanocomplex	High	Clinical prostate cancer screening	Chen et al. (2025d)

SERS has become a transformative tool in the field of biomedicine. Its research objects mainly center on disease-related biomarkers (such as proteins, nucleic acids, exosomes, etc*.*), diagnostics targeting the smallest detection units (cells or microorganisms), and liquid biopsies or tissue biopsies performed using clinically acquired body fluids or tissues. The SERS technology can adopt any strategy to adapt to different detection systems and can integrate a variety of materials and technologies (such as microfluidics, hydrogels, etc*.*) to meet the testing requirements of the aforementioned sample types.

Blood, urine, and saliva are the most frequently analyzed samples among biological fluids. These samples are easy to collect and highly clinically relevant. SERS analysis of biological fluids can be conducted through direct and indirect detection methods, enabling the identification of specific biochemical characteristics or biomarkers. These biomarkers reflect the pathophysiological features of infectious diseases, inflammation, metabolic disorders, and cancer, as well as the body’s response to treatment (Cialla-May et al., 2024). Xiao et al. designed a competitive immunoassay method that combines two zero-background SERS tags with magnetic separation technology to detect stimulants in urine (Cialla-May et al., 2024; Xiao et al., 2024). Deng et al. constructed a paper-based SERS platform, which enables on-site rapid detection of prohibited drugs in urine by pattern-printed plasmonic nanoparticles (Deng et al., 2024).

#### 2.2.1 Blood samples

Non-invasive cancer detection using human blood is a common testing method, as it can provide basic biochemical information about the human body for diagnostic purposes. Proteins and DNA in human blood originate from the death of normal blood cells in healthy individuals. However, the processes of cell apoptosis and necrosis in cancer patients can cause changes in biological macromolecules such as proteins and DNA. These altered molecules, known as tumor-associated biomarkers, provide signals for early diagnosis and facilitate the development of treatment plans. Both serum and plasma in blood contain biomarkers, including various proteins, antibodies, and exosomes released by tumor tissues into the circulatory system. Nevertheless, plasma is generally more stable and has better reproducibility than serum, because blood coagulation can lead to changes in protein levels and the activation of certain pathways.

Taking gastrointestinal tumors as an example, a SERS serum detection study based on a portable Raman spectrometer conducted serum analysis on 53 patients with gastrointestinal tumors and 25 control subjects in a real clinical setting. The results showed that there were significant differences in the peaks related to carotenoids and purine metabolites (uric acid, xanthine, hypoxanthine) in the SERS spectra between the tumor group and the control group. A diagnostic accuracy of approximately 77% was achieved using a principal component analysis combined with quadratic discriminant analysis (PCA-QDA) model. By further integrating inflammatory biomarkers in blood (such as C-reactive protein, neutrophil count, platelet count, and hemoglobin level), the diagnostic accuracy was improved to 83.33%, demonstrating great potential for the integration of SERS with clinical indicators (Avram et al., 2020).

Nguyen et al. demonstrated an early detection method for breast cancer based on a bottom-up strategy and constructed a three-dimensional gold nanocluster SERS platform. In this study, plasma samples from healthy individuals and patients were collected for further detection. The acquired SERS spectra successfully classified cancer subjects and healthy subjects into two categories, with an accuracy rate as high as 93%. These results highlight the potential of the three-dimensional plasmonic clustered SERS platform in early breast cancer detection and open up promising avenues for future research in this field (Nguyen et al., 2024).

Chen J. et al. (2025) achieved label-free SERS qualitative and quantitative analysis of serum tumor biomarkers using an attention scale fusion network, further advancing AI-assisted diagnostic accuracy. Song et al. developed a SERS method to analyze serum samples from patients with urinary system cancers (including bladder cancer, kidney cancer, and prostate cancer). By introducing iodide ions and aluminum ions, SERS spectra were collected from aggregated silver nanoparticles (AgNPs). The long short-term memory (LSTM) algorithm was used to distinguish the SERS spectra acquired from the serum of cancer patients, successfully achieving clear classification of the three types of urinary system tumors (Chen J. et al., 2025; Song L. et al., 2025).

Lee et al. utilized a SERS chip based on Au-ZnO nanostructures to obtain the spectra of blood and urine from rats with bilateral renal ischemia. Partial Least Squares-Discriminant Analysis (PLS-DA) was employed to determine the renal function evaluation rates in blood and urine, with accuracies of 99.3% and 99.9%, respectively. In another study, researchers achieved early, accurate, label-free, and non-invasive diagnosis of bladder tumors by analyzing biological metabolites in a single drop of urine using SERS. In the rat bladder cancer model, the samples were categorized into three groups: non-cancerous, early-stage cancer, and polypoid cancer. In this study, a single drop of urine from each group of samples was placed on a gold-coated zinc oxide nanoporous chip. SERS was used to selectively enhance the Raman signals of nanoscale analytes, resulting in an accuracy of ≥99.6% for both early-stage and polypoid bladder tumors. In the rat model, the Area Under the Curve (AUC) was greater than 0.996, indicating that the SERS-based diagnostic method has promising application prospects (Lee et al., 2022; Lee et al., 2024).

In research on other types of diseases, Zhang et al. prepared a new type of silver nanosheet with a nanoscale grooved structure as a SERS substrate. This detection platform can directly acquire the Raman fingerprints of monosaccharide and polysaccharide molecules, and the versatility and specificity of the platform were evaluated using machine learning (ML) and heatmaps. The application of this platform can be extended to body fluid samples, enabling non-destructive capture of glucose signals in peripheral blood, urine, sweat, and tears. This platform provides a new strategy for continuous glucose monitoring and non-invasive screening of diabetes or other related diseases (Sun et al., 2022). Li D. et al. (2022) developed a SERS method for direct detection of proteins in body fluids under native conditions, avoiding complex sample pretreatment steps (Huang et al., 2025). demonstrated colorimetric/SERS dual-mode detection of *Staphylococcus aureus* in samples from sepsis patients using Au@AgPt nanozyme arrays, showcasing the potential of SERS in clinical infectious disease diagnosis. Huang et al. developed a novel SERS-in-a-capillary platform for diagnosing heparin-induced thrombocytopenia (HIT) from the serum of post-operative patients. A uniform layer of silver nanoparticles (AgNPs) was deposited on the inner wall of the capillary via precursor deposition and heating. SERS measurements in the capillary were performed on serum samples obtained from 34 post-operative patients, and a leave-one-out cross-validation (LOOCV) classification model was developed. This model exhibited high diagnostic capability (Huang et al., 2020). Li et al. developed a SERS method for direct detection of proteins in body fluids under native conditions, avoiding complex sample pretreatment steps (Li D. et al., 2022).

#### 2.2.2 Urine samples

Urine contains a variety of substances, some of which are filtered from the circulation—such as metabolic wastes and small proteins secreted by different cell types—as well as larger proteins and cells derived from the urinary system downstream of glomerular filtration. Urine has proven to be a promising and reliable non-invasive source of cancer biomarkers. Compared with plasma or serum, urine offers several advantages for early diagnosis: it can be collected in large quantities easily, is non-invasive, and allows safe direct contact with the human body; it undergoes no significant proteolytic degradation; its composition is simpler than that of plasma/serum, which minimizes interference in urine separation and facilitates the discovery of novel biomarkers.

**FIGURE 2:**
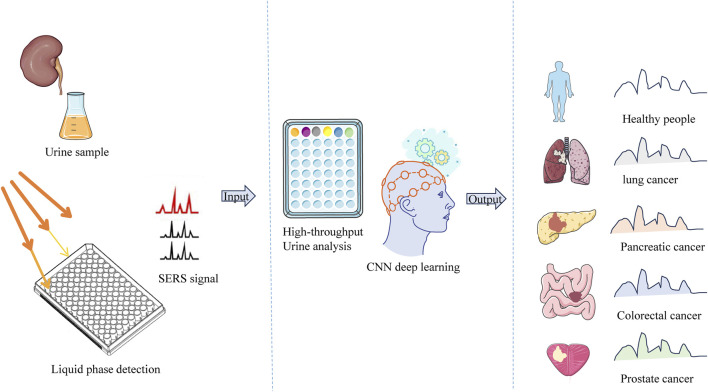
Schematic of the SERS-CNN platform for human urine sensing and multicancer diagnosis.

Al Ja’farawy et al. developed a three-dimensional gold nanoarchitecture composed of gold nanoplates/gold nanoparticles/gold nanosponges. Fabricated on a 96-well plate, this nanoarchitecture is used for label-free SERS sensing of human urine in a liquid state. The SERS well plate can be applied to detect human urine samples—including normal urine and urine from patients with various cancer types (as shown in Figure 2)—without any pretreatment (using whole urine). This study identified potential disease-related metabolites such as tryptophan and xanthine. A logistic regression model was successfully used to classify normal urine and urine from cancer groups, while a CNN-based (Convolutional Neural Network-based) model achieved multi-classification of normal urine and urine from four cancer types, yielding high clinical sensitivity, specificity, and accuracy. This study demonstrates that the developed platform can serve as a candidate for rapid, on-site, and high-throughput urine screening, which can be applied to disease diagnosis (Al Ja’farawy et al., 2024).

#### 2.2.3 Saliva and cerebrospinal fluid samples

Recently, a growing body of evidence has shown that saliva, as a novel and revolutionary target for liquid biopsy, possesses diagnostic and prognostic value. Saliva is a complex biological fluid containing a variety of proteins, DNA, miRNAs, metabolites, and microbiota. As a diagnostic method, compared with blood and tissue, saliva offers numerous biochemical advantages, such as non-invasiveness, ease of storage, and cost-effectiveness in collection. Additionally, saliva can be used for dynamic monitoring, reducing patient discomfort. Saliva-based molecular diagnosis reflects the physiological state of the human body, thus providing opportunities for monitoring oral conditions and diseases (Rapado-González et al., 2020). Tang et al. accurately distinguished between benign and malignant thyroid nodules using SERS technology in human saliva samples, and verified the results through metabolomic methods. The variation trend of metabolites in the SERS metabolic profiles was consistent with that identified by mass spectrometry, achieving a sample recognition accuracy of 95% (Tang et al., 2025).

The detection of pathogens in cerebrospinal fluid (CSF) is the gold standard for diagnosing various types of meningitis. Song et al. proposed, for the first time, a simple and reliable SERS platform for the diagnosis and identification of various meningitis. By simply mixing a silver substrate with cerebrospinal fluid samples, a meningitis classification model was established based on the fusion of spectral features between characteristic peaks and baselines. Through algorithm optimization, the classification accuracy reached 99% for autoimmune encephalitis, Cryptococcus neoformans meningitis, viral meningitis, and tuberculous meningitis (Song Y. et al., 2025).

#### 2.2.4 Exosome samples

Exosomes establish connections with the tumor microenvironment by transporting proteins and nucleic acids, and have become new biomarkers for early cancer diagnosis, monitoring, and therapeutic efficacy evaluation. Enzyme-Linked Immunosorbent Assay (ELISA) is the gold standard for exosome quantification; however, the quantification and phenotypic research of exosomes are limited due to difficulties in capturing all target subpopulations and rapidly analyzing multiple samples (Ramya et al., 2021).

Ho et al. introduced a novel droplet microfluidic platform integrated with a SERS-based sensitive sensor, which enables rapid and sensitive detection of exosomes with HER2 overexpression derived from tumor cells (Ho et al., 2024), demonstrating the platform’s potential for point-of-care testing applications. a SERS-based detection platform for circulating tumor cells. Zhang et al. leveraged the advantages of Au@Ag nanoparticles in the electromagnetic enhancement of SERS and the high specific surface area of graphene oxide for DNA adsorption, thereby achieving the detection of breast cancer-derived exosomes (Zhang et al., 2023). Su et al. developed a paper-based SERS-vertical flow sensor for the quantitative analysis of multiple exosomal proteins in serum samples (Su et al., 2023).

In digestive tract tumors, researchers developed a SERS aptasensor based on a gold nanostar-modified molybdenum disulfide (MoS_2_) nanocomposite. This sensor can sensitively detect exosomes from patients with gastric cancer, with a limit of detection as low as 17 particles/μL, demonstrating excellent stability and clinical applicability (Pan et al., 2022).

Veliz et al. employed SERS and mass spectrometry techniques to isolate and analyze exosomes from plasma samples obtained from umbilical cord blood, healthy donors, and patients with early-stage high-grade serous carcinoma. Multiple algorithms, such as principal component analysis (PCA), logistic regression, random forest, and neural networks, were used to distinguish the SERS spectra of different types of exosomes. This study enabled the diagnosis of early-stage high-grade serous carcinoma (Veliz et al., 2024).

For the label-free SERS technology using silver core-shell nanocube composite substrates, researchers analyzed lung cancer-derived exosomes by combining it with the deep learning model MobileNet V2. This approach achieved a classification accuracy of 95.23% and an AUC value exceeding 0.95, indicating that the method has a strong ability to distinguish non-small cell lung cancer cell lines. In addition, this technology enables accurate diagnosis of different subtypes of lung cancer, demonstrating the application prospects of SERS combined with artificial intelligence in clinical liquid biopsy for lung cance (Chen X. et al., 2025).

Liu et al. combined SERS to conduct label-free analysis of serum exosomes from 643 participants, aiming to clarify lung-related biochemical disorders and unique phenotypes. They used iodine-modified silver nanofilms for exosome detection, and the constructed diagnostic model achieved an accuracy of 100% for stage I lung adenocarcinoma and 81% for its precancerous lesions, respectively (Zhang D. et al., 2025). In addition, the exosome SERS model can effectively identify different subtypes and disease stages, providing guidance for precise treatment (Liu et al., 2024). Zhang D. et al. (2025) further developed a proteomics-empowered microfluidic-SERS immunoassay for the identification and detection of micropapillary lung adenocarcinoma biomarkers.

In the liquid biopsy of breast cancer, SERS technology also demonstrates excellent performance. In a study that used a magnetic SERS platform to achieve continuous isolation and signal enhancement of breast cancer-derived exosomes, principal component analysis (PCA) was employed to successfully distinguish exosomes derived from two types of cancer cells, namely, MCF-7 and MDA-MB-231. The sensitivity and specificity for diagnosing breast cancer patients and healthy individuals reached 91.67% and 100%, respectively. This platform does not require complex pretreatment or signal enhancers, and has the advantages of real-time and efficient detection, making it suitable for clinical applications (Li et al., 2021).

Jia et al. used SERS to analyze the molecular changes in serum exosomes of HER2-positive breast cancer patients before and after neoadjuvant therapy, and developed a prediction model with an AUC value exceeding 0.89. The potential of this system in accurately predicting early treatment responses provides insights for evaluating treatment regimens for HER2-positive breast cancer (Jia et al., 2025).

**FIGURE 3:**
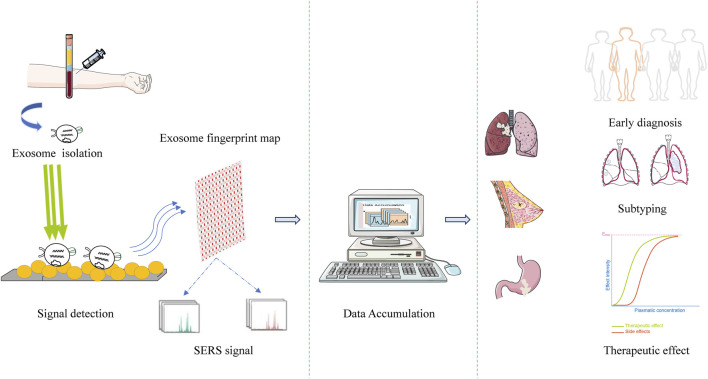
SERS-based exosome detection for identifying the origin of tumor tissues.

In the study on exosomes derived from cancer stem cells, researchers constructed a self-functionalized three-dimensional nanosensor, which achieved ultra-sensitive detection of exosomes at an extremely low concentration (10 particles/10 μL). Combined with an artificial neural network, this sensor exhibited 100% sensitivity and specificity in the diagnosis of breast cancer, lung cancer, and colorectal cancer. It could also accurately distinguish the origin of tumor tissues (as shown in Figure 3), breaking through the biological and technical bottlenecks faced by traditional liquid biopsies (Haldavnekar et al., 2022a; Haldavnekar et al., 2022b).

#### 2.2.5 Circulating tumor cells (CTCs)

Circulating Tumor Cells (CTCs) contain disease information consistent with the tumor, and researchers conduct efficacy evaluation based on the types of CTCs and the changing trends of their quantities (Fabisiewicz and Grzybowska, 2017). Reza et al. designed a microfluidic device based on SERS detection to screen dynamic single CTCs, and clarified the heterogeneous expression of multiple protein markers in response to treatment. Li et al. further developed a SERS labeling method for multiplexed cell surface immunophenotyping analysis, which was evaluated and validated in two different cell models (red blood cells and breast cancer cells) (Reza et al., 2021; Li J. et al., 2022).

For the detection of circulating tumor DNA (ctDNA), researchers have adopted a method that combines enzymatic cyclic amplification with gold nanoparticle-assisted Surface-Enhanced Raman Scattering (SERS) technology. This approach enables the sensitive detection of ctDNA from diffuse intrinsic pontine glioma (DIPG), with a limit of detection (LOD) as low as 9.1 FM, and can accurately identify single-base mutation sequences. It provides strong technical support for early diagnosis and therapeutic efficacy monitoring in precision medicine.

Through the combination of magnetic enrichment and silver nanoclusters, the enhancement of SERS signals is promoted, thereby achieving accurate quantification of the methylation level of tumor suppressor genes. This method offers a highly sensitive and quantitative detection strategy for early-stage tumor liquid biopsy (Chen D. et al., 2025; Miao et al., 2021). In another study, a SERS-based detection platform for circulating tumor cells (CTCs) was developed. By integrating multiple recognition molecules and machine learning, the detection sensitivity reaches one to two cells per milliliter, which significantly improves the accuracy of early tumor monitoring (Zhang C. et al., 2025; Sánchez-Castillo et al., 2024). Chen D. et al. (2025) developed dual-enhanced SERS immuno-nanocomplexes for PSA-mediated PHI assay, demonstrating application potential in clinical prostate cancer screening.

In summary, Surface-Enhanced Raman Scattering (SERS) technology, with its ultra-high sensitivity, specificity, and multiplex detection capability, is constantly expanding its applications in liquid biopsy, covering a variety of tumor types (such as gastrointestinal tumors, lung cancer, breast cancer, glioma, osteosarcoma, etc*.*). Combined with artificial intelligence methods like deep learning, SERS has significantly improved the accuracy of diagnosis and clinical practicality. In the future, with the innovation of nanomaterials, the optimization of signal enhancement strategies, and the advancement of data analysis technologies, SERS is expected to enable rapid, accurate, and non-invasive diagnosis in liquid biopsy, providing important technical support for the early detection of tumors and personalized treatment.

## 3 Discussion

### 3.1 Current main challenges of SERS technology

Despite the remarkable potential of SERS technology in tumor liquid biopsy, several critical challenges must be addressed before its widespread clinical adoption. These challenges span the entire analytical workflow—from the design and fabrication of SERS substrates to the interpretation of spectral data—and are particularly pronounced when transitioning from controlled laboratory settings to complex clinical environments. Two interrelated aspects stand out as the most pressing hurdles: the standardization and reproducibility of SERS measurements, and the biocompatibility and safety of SERS nanoprobes. Addressing these challenges requires a multifaceted approach that integrates advances in nanomaterial engineering, surface chemistry, and analytical methodology, as discussed in the following subsections.

#### 3.1.1 Standardization and reproducibility

First, although sensitivity is one of the core advantages of SERS, the stability and reproducibility of signals in complex biological samples are limited by the substrate material and measurement conditions. The shape, size, composition, and arrangement of nanomaterials have a significant impact on the signal enhancement effect of SERS. For instance, noble metal nanoparticles (such as silver and gold), nanowires, and nanostars have been extensively studied to improve the signal enhancement factor (Avram et al., 2020). However, the complex matrix effects and non-specific adsorption in biological samples may lead to an increase in signal noise, thereby reducing the detection sensitivity (Li et al., 2021). The fluctuation of SERS signals is further exacerbated by the inhomogeneous distribution of hot spots, which forms a bottleneck for sensitivity improvement (Dey, 2023).

Secondly, the specificity issue is mainly reflected in the insufficient selective binding between the target molecules and the SERS-active substrate. Although nanoparticles modified with biofunctionalization such as antibodies, aptamers, and molecularly imprinted polymers (MIPs) have significantly improved specificity and selectivity, there is still the possibility of background interference and non-specific binding in complex body fluids (Hou et al., 2020; Wei et al., 2025). For example, although the multiplex-labeled SERS probes used for circulating tumor cell (CTCs) detection exhibit high sensitivity, they still need to overcome the interference from other cells and proteins in the complex blood environment 13. Furthermore, the expression heterogeneity of tumor markers also poses a challenge to specific detection, requiring SERS probes to have the ability to recognize multiple markers in order to improve the accuracy of diagnosis (Wu et al., 2024).

Thirdly, biocompatibility and safety issues are key obstacles to the clinical application of SERS technology. Many high-performance SERS substrates are made of silver nanomaterials; although they exhibit excellent signal enhancement effects, the release of silver ions and the generation of reactive oxygen species (ROS) may trigger cytotoxicity and inflammatory responses (He et al., 2021). To address this, researchers have adopted strategies such as protein coating and polyethylene glycol (PEG) modification to improve the biological stability of nanoprobes, reduce their toxicity, and simultaneously enhance their targeting ability (Srivastava et al., 2022). In addition, the metabolic property and *in vivo* clearance capability of materials are also important considerations in the design of biocompatible SERS probes. For instance, gold nanoparticles coated with copper sulfide can accelerate *in vivo* metabolism and excretion, thereby reducing the risk of long-term accumulation (Zhang et al., 2024).

In addition, sample preparation and interference from complex matrices are also bottlenecks restricting the application of SERS technology. Various components in biological samples may lead to spectral overlap and signal interference, while traditional sample processing methods are cumbersome and time-consuming, making it difficult to meet the needs of rapid clinical diagnosis (Lai et al., 2022). To address this issue, integrated sample pretreatment technologies and machine learning-assisted spectral data analysis methods have been gradually developed to achieve rapid, accurate, and high-throughput detection (Lai et al., 2022; Rojalin et al., 2022).

The translation of SERS technology from research laboratories to clinical practice is fundamentally hindered by issues of standardization and reproducibility. These challenges originate at multiple levels of the analytical workflow. At the substrate level, the synthesis of metallic nanoparticles—whether gold, silver, or core-shell structures—is subject to batch-to-batch variations in size, shape, and morphology, all of which critically influence the density and intensity of electromagnetic “hotspots” (Le Ru and Auguié, 2024). Even minor deviations in synthesis parameters can lead to significant differences in signal enhancement factors, compromising the comparability of results across experiments and laboratories (Dey, , 2023). At the measurement level, the lack of universally adopted protocols for laser wavelength, power density, integration time, and sample preparation further exacerbates signal variability. The stochastic distribution of hotspots across the substrate surface means that spectra acquired from different regions of the same sample can vary substantially, making quantitative analysis particularly challenging. To address these issues, several strategies have been explored. The incorporation of internal standards, such as Raman reporter molecules with stable and distinct signals, enables ratiometric measurements that correct for signal fluctuations (Rojalin et al., 2022). Advances in nanofabrication techniques, including self-assembly and templated lithography, have enabled the production of large-area substrates with more uniform hotspot distributions (Bauman et al., 2022). More recently, machine learning algorithms have been employed to recognize and correct for spectral variations, improving the robustness of classification models across different batches and experimental conditions (Lai et al., 2022). Despite these efforts, achieving the level of reproducibility required for clinical diagnostics—where inter-laboratory and inter-instrument comparability is essential—remains an ongoing challenge that demands collaborative efforts toward establishing industry-wide standards.

#### 3.1.2 Biocompatibility and in vivo safety

While the performance of SERS nanoprobes is paramount, their biocompatibility and safety profile are equally critical for clinical translation, particularly for *in vivo* applications. This concern is most pronounced for silver-based nanomaterials, which offer exceptional plasmonic properties but pose inherent toxicity risks. The primary toxicity mechanisms involve the release of Ag^+^ ions and the generation of reactive oxygen species, which can induce oxidative stress, mitochondrial dysfunction, DNA damage, and inflammatory responses in exposed cells (Hou et al., 2020). These effects are concentration- and time-dependent, raising concerns about both acute toxicity and long-term accumulation in organs such as the liver, spleen, and kidneys. To mitigate these risks, various surface modification strategies have been developed. Coating silver nanoparticles with a thin gold shell (Au@Ag core-shell structures) not only preserves or enhances SERS activity but also significantly reduces Ag^+^ release by acting as a physical barrier (Tiryaki et al., 2024). Surface functionalization with polyethylene glycol improves colloidal stability, reduces protein corona formation, and minimizes non-specific interactions with immune cells, thereby prolonging circulation time and reducing immunogenicity (Wei et al., 2025). More advanced approaches involve biomimetic coatings, such as red blood cell membranes, which enhance biocompatibility and enable immune evasion while preserving the targeting functionality of the nanoprobe (Wei et al., 2025). Beyond surface modification, the design of nanoparticles with controlled clearance pathways has emerged as a key strategy. For instance, Au@CuS core-shell nanostructures have been shown to undergo accelerated hepatobiliary excretion, reducing the risk of long-term tissue accumulation and toxicity (He et al., 2021). Looking forward, systematic *in vivo* studies are needed to fully characterize the biodistribution, metabolism, degradation, and elimination of SERS nanoprobes over extended time periods. The establishment of regulatory guidelines specific to nanomedicine will be essential to ensure that these promising diagnostic tools can be safely translated to clinical use.

In summary, although current SERS technology has the advantage of high sensitivity, it still needs to address challenges in aspects such as signal stability, detection specificity, biocompatibility, and sample complexity for practical tumor diagnosis. Future research should focus on the design of SERS probes with high performance, low toxicity, and multi-recognition capabilities, and combine advanced data processing and sample pretreatment technologies to promote the wide application and translation of SERS technology in clinical tumor diagnosis (Chen et al., 2025d).

### 3.2 Future research directions and solutions

As an ultra-high-sensitivity molecular detection method, Surface-Enhanced Raman Scattering (SERS) technology has shown great application potential in the field of tumor diagnosis. One of the key focuses of future research is the development of novel probes and the optimization of nanomaterials to further enhance the sensitivity, selectivity, and biocompatibility of SERS technology, thereby meeting the needs of clinical diagnosis and treatment.

First, the design of novel SERS probes tends to integrate multifunctionality and intelligent responsiveness. For example, responsive SERS probes can generate detectable changes in Raman signals based on variations in specific metabolites or enzymes in the tumor microenvironment, enabling dynamic monitoring of the tumor metabolic state. Literature has reported a stimulus-responsive SERS probe that can perform ultra-high-sensitivity and high-specificity imaging of tumor-related metabolites, providing support for tumor surgery guidance and molecular typing in liquid biopsie (Yin et al., 2022). In addition, significant progress has also been made in using SERS probes to detect the activity of specific enzymes in the tumor microenvironment (such as matrix metalloproteinase MMP-2). By constructing a ratiometric SERS sensing system with internal standards, accurate quantification of MMP-2 activity and differentiation of tumor cells have been achieved (Zhu et al., 2025).

The optimization of nanomaterials is key to enhancing SERS performance. Traditional metal nanoparticles (such as silver and gold) are widely used due to their excellent localized surface plasmon properties, but their biocompatibility and stability still need improvement. Recent studies have employed bio-inspired strategies: coating SERS nanoparticles with red blood cell membranes significantly enhances their dispersibility, signal intensity, and tumor-targeting function (Srivastava et al., 2022). In addition, the construction of composite nanostructures—such as Au@CuS core-shell nanoparticles—not only improves the signal-to-noise ratio of SERS signals but also exhibits excellent photothermal conversion performance. This enables image-guided photothermal therapy for tumors, accelerates the hepatobiliary metabolic clearance of nanoparticles, and reduces toxicity risks (Zhang et al., 2024). Furthermore, pure semiconductor SERS-MRI dual-modal nanoprobes based on ferrite (Fe_3_O_4_) nanoparticles have demonstrated high-sensitivity *in vitro* and *in vivo* tumor imaging capabilities, expanding the functional scope of SERS nanomaterials.

In terms of the detection of tumor cells and circulating tumor cells (CTCs), multi-color encoded nanoprobes combined with microfluidic chip technology have enabled efficient enrichment and simultaneous detection of multiple tumor markers, significantly improving the sensitivity and specificity of diagnosis (Kedarisetti et al., 2020). Meanwhile, dual-enhanced SERS probes that combine nanostar-shaped metal structures with physical enhancement technology have successfully achieved highly sensitive localization of *in vitro* tumor cells and stable acquisition of signals, demonstrating potential clinical application value (Zhao et al., 2022). In the direction of point-of-care testing (Zhi et al., 2025), developed a lateral flow immunoassay platform based on multimetallic intra-nanogap nanozymes, achieving ultrasensitive multimode detection of pathogens in clinical samples, providing new insights for the integration of SERS with POCT technologies.

In the future, the development of SERS probes still needs to address the interference caused by the complexity of biological samples, improve the repeatability and stability of signals, and integrate artificial intelligence and machine learning algorithms to realize the automated analysis of SERS data and early tumor diagnosis (Zhang C. et al., 2025; Shi et al., 2024). In addition, the safety, degradability of nanomaterials and the optimization of metabolic pathways are also key focuses of future research, aiming to develop non-toxic and long-term stable SERS nanoprobes for clinical translation. For instance, the design of novel degradable nanoreactors not only enables tumor microenvironment-triggered synergistic therapy but also monitors the therapeutic process through SERS feedback, which reflects the development trend of multifunctionalization of nanomaterials (Zhang et al., 2024). To summarize, the future development of tumor diagnostic probes based on Surface-Enhanced Raman Scattering (SERS) will focus on multiple directions, including intelligent responsiveness, precise design of nanostructures, improvement of biocompatibility, and integration of multiple functions. Through interdisciplinary integration, SERS technology will be promoted to move toward precise, rapid, and non-invasive clinical applications.

### 3.3 Integration of precision medicine and personalized therapy

In recent years, Surface-Enhanced Raman Scattering (SERS) technology has demonstrated significant application potential in the field of precision medicine due to its extremely high sensitivity and specificity, and it plays a crucial role especially in personalized tumor therapy. By optimizing the design of nanoprobes, SERS has achieved targeted functionalization, improved biocompatibility, and integration with multimodal imaging systems. This effectively overcomes the limitations of traditional imaging technologies in terms of sensitivity, temporal resolution, and spatial resolution, enabling the accurate capture and dynamic tracking of molecular events in the *in vivo* biological environment. This advantage allows SERS to provide real-time and *in situ* molecular information in aspects such as early tumor diagnosis, surgical navigation, drug delivery monitoring, and dynamic pathological analysis, thereby greatly enhancing the precision and personalization level of tumor diagnosis and treatment (Chen et al., 2025e).

In personalized treatment strategies, SERS is not only used for early diagnosis and pathological typing, but also combined with nanotechnology to develop multifunctional nanocarriers, enabling the integration of diagnosis and treatment. For example, nucleic acid conjugates based on graphene-gold nanoparticles (Au@GO NP-NACs) achieve highly sensitive detection of tumor cells through SERS, while integrating photothermal therapy, gene therapy, and chemotherapy to form a multimodal synergistic treatment platform. This platform can implement precise killing of different types of tumor cells and achieve personalized targeted therapy. Both *in vitro* and *in vivo* experiments have demonstrated its superior performance in cancer diagnosis and treatment, providing a new technical pathway for personalized tumor therapy in precision medicine (Yang et al., 2021).

In addition, by combining deep learning and microfluidic technology, SERS enables efficient capture and classification of tumor cell exosomes, and exhibits extremely high accuracy especially in the early diagnosis and molecular typing of non-small cell lung cancer (NSCLC) subtypes. This method uses polystyrene microspheres combined with gold nanocubes and anti-CD-9 antibodies to construct capture units, and achieves accurate identification of different lung cancer subtypes through SERS signal detection combined with deep learning algorithms. This technology overcomes the invasiveness and limited information of traditional tissue biopsy, provides a more convenient and efficient personalized diagnosis and treatment tool for liquid biopsy, and further promotes the development of precision oncology (Chen H. et al., 2025).

In summary, relying on its high-sensitivity molecular detection capability, the development of multifunctional nanomaterial carriers, and the integration with artificial intelligence, SERS technology has successfully achieved a high level of personalization and precision in tumor diagnosis and treatment. In the future, with the continuous improvement of signal stability, multimodal imaging collaboration, and data standardization processing methods, SERS will play a more significant role in clinical translation, driving personalized tumor therapy into a new era of intelligent and integrated diagnosis and treatment (Chen et al., 2025e).
